# Supplementary material for: Are carbon emissions trading and green financial instruments synergistic? -Comprehensive quantitative research based on content analysis
Source: PLoS One. 2024 Mar 7;19(3):e0298601. doi: 10.1371/journal.pone.0298601 (PMC10919601; doi:10.1371/journal.pone.0298601)
Supplement: S1 Data — (ZIP) [file pone.0298601.s001.zip › Data/地级市Z_F工作报告.zip!/╜¡╬≈/─╧▓2╩╨/└┤╫╘╣1⁄2╓┌║┼í╛┬φ┐╦╩2╛▌═°í┐_2019.docx]

各位代表：

现在，我代表市人民政府向大会报告工作，请予审议，并请各位市政协委员和列席会议的同志提出意见。

一、2018年工作回顾

过去一年，在省委、省政府和市委的坚强领导下，全市上下深入学习贯彻习近平新时代中国特色社会主义思想和党的十九大精神，从更高层次贯彻落实习近平总书记对江西工作的重要要求，牢牢把握高质量跨越式发展首要战略，按照省委提出的“二十四字”工作方针，狠抓各项工作落实，较好完成了市十五届人大三次会议确定的目标任务。

——综合实力持续增强。完成财政总收入938.6亿元，按省口径计算增长11.1%；地方一般公共预算收入461.7亿元，增长10.7%。南昌县、西湖区、红谷滩新区、高新区财政总收入超百亿。年末金融机构本外币存贷款余额分别为10733.1亿元、12124.6亿元，增长5.9%、17%，占全省比重30.4%、39.7%，存贷比113%。预计地区生产总值增长8.8%左右；规模以上工业增加值增长9.4%左右；500万元以上固定资产投资增长11%左右；实际利用外资增长15.1%；实际利用内资增长19.2%；社会消费品零售总额增长11.1%左右；城镇和农村居民人均可支配收入分别增长8.3%、9%。

——发展质量稳步提升。财税收入结构持续优化，全市税收收入占财政总收入和地方一般公共预算收入的比重分别达到89.6%、80.5%，高于全省8.3和10.4个百分点。我市地方一般公共预算收支的增幅均在全省设区市排列第一。工业经济效益持续改善，全市规模以上工业主营业务收入增长15%左右，利润总额增长18%左右，人均全员劳动生产率达到41万元左右。非公经济活力不断释放，非公经济增加值占全市比重达57%左右。万元GDP能耗下降3%，万元规模以上工业增加值能耗下降2%。

——城市影响力不断扩大。首届世界VR产业大会举世瞩目，习近平总书记专门发来贺信，为全市推动新技术新产品新业态新模式发展增强了信心。“八一”军体运动队集体落户南昌，29支运动队的37个项目队身披“八一南昌”战袍征战国内外赛场，6个全国职业联赛主场落户南昌。成功举办第五届世界绿色发展投资贸易博览会、首届国际手机零配件展览会、第四届全国民宿客栈旅游投资南昌峰会、全国文房四宝艺术博览会、南昌国际马拉松等大型活动，“天下英雄城•南昌”城市名片更加响亮。

一年来，我们重点抓好了以下工作：

（一）聚焦振兴实体经济，现代化产业体系加快建设。

“一核两重”产业加速发展。现代制造业稳中提质。全年新增规模工业企业328户、净增290户左右。规模以上工业增加值增幅连续18个月排名全省第一。战略性新兴产业增加值增长11%左右，占规模以上工业增加值比重达28%左右。汽车和新能源汽车、绿色食品、电子信息产业主营业务收入分别突破1300亿元、1100亿元和1000亿元。江铃集团挺进“千亿俱乐部”。新增1个百亿企业、3个五十亿企业。移动智能终端企业超50家，可实现90%以上的手机零部件本地配套；LED产业在全国率先形成上中下游完整产业链。汽车及零部件企业超200家，形成了完整的产业体系。中医药科创城“两核”建设有序推进，江中集团科研中心项目投入使用。航空城建设加速推进，国产C919大型客机在瑶湖机场转场试飞成功，全国首个省局共建的江西适航审定中心、北航江西研究院、中国商飞ARJ21生产完工交付中心纷纷落户。现代服务业稳中趋优。全国服务业综合改革试点、全国物流标准化试点取得新进展。新建区新城吾悦广场、青山湖区万达广场、经开区儒乐湖健康文旅产业小镇、安义县安南文旅小镇、青云谱梦想小镇、红谷滩新区红星美凯龙全球家居生活广场等一批商业综合体相继建成。西湖区入选中国楼宇经济十大活力城区。预计全市完成电子商务相关交易额3000亿元，增长34.9%，全国电商前五强齐聚向塘铁路物流港。进贤县荣获全国电商示范百佳县。旅游产业再次实现“双突破”，全年接待旅游总人数1.51亿人次、旅游综合收入1520亿元。汉代海昏侯国遗址公园、京台民俗文化大院、泊园茶村旅游综合体等重大旅游项目快速推进。滕王阁景区创5A成功，实现我市5A级景区零的突破；李渡烧酒作坊遗址景区获评国家4A级旅游景区；湾里区获评省级全域旅游示范区，梅岭风景区获评中国体育旅游精品景区；黄马乡、文港镇获评省级旅游风情小镇。会展业呈井喷发展态势，全国性展会数量和面积实现翻番。现代农业稳中向好。预计完成农林牧渔及服务业总产值320亿元左右，增长3.3%左右。全年粮食总产量47.27亿斤，实现“十五连丰”。大力实施“一乡一园”建设，新增现代农业示范园45个。全国农田建设现场会在我市召开。中国云厨南昌全域产业园、铭宸智慧农业科技园等重大项目相继落户并开工建设；农文旅结合、镇村园联动的25个田园综合体全面启动，太平九龙溪、石鼻千年古村等田园综合体基本建成，黄马凤凰沟入选全国首批田园综合体建设项目；赣鄱珍奇植物园PPP项目入选全国第四批PPP示范项目。农业产业化稳步推进，新型农业经营主体蓬勃发展，国家级农业产业化龙头企业、农民专业合作社、家庭农场数量继续走在全省前列，9家企业位列中国农业产业化龙头企业500强。凌继河被评为全国十佳农民。打造了68个“一村一品”市级示范点。

创新驱动战略深入实施。成立科技创新领导小组，设立科技发展引导母基金，吸引社会资本组建子基金，采取股权投资方式支持创新发展。平台不断拓展。南昌科技广场、红谷滩科创小镇、航空科创城、南昌国家大学科技城以及中科院苏州纳米所南昌研究院、天津大学南昌微技术研究院、哈工大机器人（南昌）智能制造研究院、中山大学（南昌）产业研究院、直接质谱与精准诊断国际转化研究中心、工业过程智能化研究院、江西省经典名方研发中心等研发机构加快建设。全省第1家、中部省份第2家国家级知识产权保护中心在我市挂牌运行。省部共建核资源与环境国家重点实验室落户经开区。全省首个中科院直属二级机构江西产业技术创新与育成中心落户高新区。国家级重点实验室、企业技术中心各新增1家，省级工程技术研究中心、重点实验室、企业技术中心分别新增8家、7家、5家，市级众创空间备案新增9家。人才加速集聚。出台“人才新政”22条，制定配套细则34个，人才落户超万人。实施“洪”字号系列人才计划，引进海内外“高精尖缺”人才137人次，柔性引进院士40人。全省首家诺贝尔奖院士工作站落户青山湖区。中国南昌人力资源服务产业园致力打造人才蓝海，帮助省内外数千家企业招聘各类人才和服务用工110万余人。我市荣获中国优化人才创新创业环境十佳城市。能力不断提升。预计全市R&D经费占GDP比重1.7%；工业技改投资增长17%左右；国家高新技术企业数量突破1000家，增长70%以上，创历史新高；全市专利授权量达13000件、发明专利申请量6370件、每万人有效发明专利拥有量9.5件，技术合同交易额达48.31亿元，增长12.9%。全国5G规模组网建设及应用示范工程、全国移动支付便民示范工程加快推进。“03专项”试点示范高位推进，全市基本实现城区、县区、工业园区NB-IoT连续覆盖，4G网络全面覆盖城市和农村，率先在全省开通首张5G实验网，物联网技术及产品应用于智慧消防、公安、环保等27个领域。

现代金融体系不断完善。防范化解重大风险攻坚战初战告捷。多渠道加强地方政府债务管理，政府债务风险管控工作稳步推进。开展“清楼扫街”行动，严厉防范打击非法集资，全面加强P2P等重点领域整治，支持设立市第一、第二金融法庭，着力消除潜在风险隐患。金融业发展成果丰硕。红谷滩全省金融商务区落户各类金融企业超1000家，江西银行成为“江西金融第一股”，江西高速财务公司落户红谷滩新区，恒邦保险新增三家省级分公司，全省首家民营银行裕民银行申报筹建获得重大进展，浙商银行南昌分行正式落户，南昌金融控股有限公司启动运作，全省首家区级金控公司获批组建，基金、担保、转贷、租赁、供应链金融等金融组织日益健全。金融支持实体经济水平明显提升。打好企业上市“映山红行动”攻坚战，实现“新增2家、控股1家、申报2家、备案2家”良好开局；打好降成本优环境持久战，持续抓好系列惠企政策落地，积极开展政银企对接活动，国有出资全覆盖融资担保体系加速形成，全市转贷公司累计提供“过桥”资金超300亿元；打好上市公司保卫战，组建首期规模20亿元的纾困基金，与上市公司共渡难关。继续推进“重资本轻资产”招商模式，综合运用多种金融工具，有力保障一批重点项目落地投产。投融资体制改革不断深入。按照“整合资源、分类实施、优化路径、分级负责”的原则，进一步深化全市产业投融资体制改革。全面开展市属国有资产清产核资、资产重组，投融资主体在市场化转型中做强做大，市政公用集团资产突破1000亿，市政控股率先获得AAA信用评级。科技金融试点初见成效，产融合作试点纵深推进。

（二）聚焦城乡融合发展，“美丽南昌•幸福家园”打响品牌。

城市建设加快推进。《南昌市城市总体规划（2016-2035）》以及城市综合交通体系、轨道交通线网、城市更新、城市双修、重要节点区域等专项规划加快编制。重点推进总投资1500亿元的城建项目68个，完成投资210亿元。昌东大道南延、志敏大道、前湖立交及前湖大道快速路主线相继通车；轨道交通2号线后通段、3号线、4号线，洪都大道快速路改造，昌九大道快速路改造二期、昌西大道、海昏大道、昌南大道快速路改造等项目快速推进；昌吉赣客专、昌景黄客专、昌九客专项目推进有关工作稳步开展。提前三个月完成21240户棚户区改造任务，54个安置房建设加快推进。“海绵城市”、城市地下综合管廊、污水管网、公共停车场建设扎实推进。省委党校、红谷隧道、中至信息大厦、北京银行南昌分行营业大楼四个项目荣获鲁班奖。

城市环境持续提升。总投资约68.5亿元的352个“六改”项目扎实推进。完成40条道路建筑立面整治和近200条道路城市家具改造，对全市8781个强弱电箱体进行了彩绘美化，实施了一江两岸灯光秀南北延伸、青山湖景区、抚河沿线、八一公园，以及中山路、胜利路、阳明路、站前路等一批景观亮化项目，打造了胜利路、广州路、火炬二路、南京东路等15条精品样板街区，高标准完成了八一大道、北京路“一纵一横”建筑立面综合改造，改造提升了西湖区朱紫巷小区、东湖区美福庭院、南昌县莲垦社区等一批老旧社区。“马路本色”行动实现全覆盖，主干道全部实行机械化清扫和“以克论净”考核，公共机构垃圾分类实现全覆盖，62个小区垃圾分类试点全面铺开，建筑施工扬尘、餐饮油烟等专项治理巩固落实，“洁净南昌”品牌全面打响。强力开展了施工围挡、出租汽车、机场火车站、客运场站、港口码头等专项整治行动，加强了智慧工地、智慧交通管理，截至2018年11月底，我市在全国100个大城市交通拥堵指数排位由2017年的41位下降至77位。

乡村环境明显改善。全市2334个新农村村点“七改三网”整治任务基本完成；开展了新农村“五拆五清”工作，共拆除各类有碍观瞻建筑160万平米。21个特色小镇、109个“五位一体”示范村、30个新户型推广示范村建设持续推进；启动了10条新农村“四精”示范线路建设。南昌县八一乡钱溪村和向塘镇剑霞村、安义县新民乡珠珞村被评为第五届全国文明村，安义县石鼻镇、南昌县幽兰镇被列为第二批省级特色小镇创建名单，安义县罗田村被授予“中国美丽休闲乡村”称号。铁路公路沿线环境综合整治、公路货运车辆超限超载治理成效明显。农村生活垃圾处理率达100%，圆满完成国家农村生活垃圾治理考核验收工作。建设集中供水工程15处，87个农村村点水环境治理加快推进。

生态环境巩固优化。打好蓝天保卫战。“四尘三烟三气两禁”专项整治持续开展，打造了“禁燃禁放”南昌模式，“南昌蓝”成为城市靓丽名片。在城区人口多、汽车多、工地多、企业多、污染物排放总量大“四多一大”的情况下，全市PM2.5年均浓度为每立方米30微克，比2017年下降26.8%，浓度值在全省最低，下降幅度排全省第一；PM10年均浓度为每立方米64微克，下降15.8%，下降幅度排全省第一；空气质量优良率为89.6%，比2017年上升7.6%，上升幅度排全省第一，连续五年在中部六个省会城市排名第一。空气质量在中部六个省会城市和全省各设区市中率先达到二级标准。打好碧水攻坚战。严格落实河湖长制，全面消灭6个劣Ⅴ类水质断面，完成6个黑臭水体整治。启动全域治水战略，九龙湖污水处理厂、南昌县污水处理厂、进贤县污水处理厂的一级A提标改造项目投入运行，启动了青山湖等5座污水处理厂一级A提标改造工作。全市地表水国考、省考断面水质状况总体稳定，集中式饮用水源地水质水量达标率为100%。东湖区贤士湖、幸福渠流域水系治理经验获得各界高度点赞。打好净土防御战。在全省率先开展重点行业企业用地污染状况调查前期工作，扎实推进全市重点行业企业、工业园及重点区域周边土壤监测，深入开展“绿盾2018”、工业固体废弃物排查整治、全市机动车维修和拆解企业危险废物规范化管理等专项行动，全市土壤环境质量总体良好。打好环保督察整改歼灭战。中央环保督察、省环保督察、鄱阳湖水环境问题专项督察反馈问题整改落实有序推进。破解了麦园垃圾处理厂整改、黑臭水体整治、蓑衣荚排污口整治等一批历史遗留问题。打好生态文明建设持久战。积极参与长江经济带“共抓大保护”攻坚行动，持续深入推进河湖长制，全面推行林长制，编制自然资源资产负债表，全市生态文明试验区建设持续加强。

（三）聚焦强动力添活力，改革开放全面纵深推进。

改革继续深化。市县机构改革顺利启动、平稳推进，退役军人事务局、大数据发展管理局等新机构加快组建。“放管服”改革持续深化，相对集中行政许可权改革试点深入推进，探索推行了“六多合一”集成审批模式，“一枚印章管审批”初显成效，审批证明材料压缩37%，审批环节压缩45%，审批时间压缩33%，行政审批事项网上可办率达80%，政务服务事项标准化“南昌模式”在全省推广；在全省率先试行政务办事大厅错时延时工作制，打造了365天“不打烊”的政务服务。企业事中事后监管改革持续推进，南昌市公共信用信息共享系统上线运行。全国首个人才服务“港网窗”一体化服务平台正式启动。建设用地使用权转让、出租、抵押二级市场试点工作通过国家自然资源部验收。加快推进开发区改革创新发展，工业园区先行开展租售并举住房制度改革工作，昌南工业园在全省率先转型为新经济产业聚集区。按照权力下放、资金下沉、重心下移、监管跟上、考核精细的思路，推进了财税管理、城市管理等体制改革。公立医院改革成效显著，公立医院药占比（不含中药饮片）同比下降4.41个百分点、医疗服务收入占比同比提高6.02个百分点。积极推进养老服务综合改革，进一步完善“互联网+大养老”服务体系，全市新建居家养老服务中心公建民营比重达62%。东湖区探索“时间银行”互助养老模式，打造了示范性养老服务品牌。稳步推进殡葬综合改革，大力开展绿色殡葬专项行动，打造了文明祭扫“南昌模式”。青云谱全国城区老工业区搬迁改造试点、湾里区全国农村集体产权制度改革试点工作成效明显。我市率先在全省全面实现财政国库集中支付电子化管理。军民融合改革、林业体制改革、粮食收储制度改革、供销合作社综合改革、户籍制度改革等各领域改革扎实推进。

开放卓有成效。积极融入“一带一路”和长江经济带，参与或举办赣京会、赣深会、赣港会、赣台会、亚布力夏季峰会等招商活动76场，共引进重大项目483个，其中100亿元以上项目6个，50亿元以上项目10个，20亿元以上项目48个。英华达智能制造、维科南昌基地、海康威视、中国商飞等一大批高质量、高科技的产业项目签约落户，欧菲未来城、乾照光电、同兴达、兆驰新增封装生产线等重大项目快速推进，中微半导体、科莱电子等42个重大工业项目当年签约、当年开工、当年投产。外贸新业态新模式蓬勃发展，南昌跨境电子商务综合试验区获批设立。南昌昌北国际机场旅客吞吐量达1352万人次，货邮吞吐量8.2万吨，增速居全国干线机场前列；海铁联运完成外贸运量1.9万个标箱，增长55%。进一步打通国际物流大通道，新开中欧（南昌—莫斯科）国际铁路货运班列，累计开行24列，货值超1亿美元，基本实现每周一班的常态化运行。获批设立昌北机场海关、赣江新区海关、青山湖海关等3个具有法人资格、功能完备的隶属海关。积极开展国际产能合作，实施优势产业海外扩张和富余产能海外转移，我市“走出去”步伐已遍及近30个“一带一路”沿线国家和地区，2家企业首次登上“全球最大250家国际承包商”榜单。城市交流合作更加深入，组织市艺术团赴日本大阪演出，我市荣获“国际友好城市交流合作奖”“中日韩优秀友城合作奖”。

（四）聚焦人民美好生活需要，社会各项事业全面进步。

脱贫攻坚取得新成效。扎实开展了“春季攻势”“夏季整改”“秋冬会战”三大行动，自查梳理的6大类101个问题已全部整改到位。完成了年度33个省市级贫困村退出和6950名贫困人口脱贫的任务。贫困村的新农村建设、贫困户的财政兜底保障实现全覆盖。扶持了18699户贫困户发展产业，覆盖率达100%；贫困户加入专业合作社比例达52.17%，2692户贫困户享有资产收益扶贫；帮助3555名未脱贫劳动力实现就业；1228所学校全面实施了义务教育扶贫资助政策学校校长与乡镇属地双负责制，全市3342名建档立卡贫困学生享受教育补助政策，资助率100%。健康扶贫实现“一个确保，五个全覆盖”，重大疾病商业补充保险由每人每年90元提高到330元。落实“一对一”结对帮扶责任人17991人，社会化大扶贫格局全面形成。

民生保障得到新加强。筹集资金约346亿元，集中办好了涉及群众切身利益的101件实事。完善了社会救助体系，在全省率先开展城镇支出型贫困低收入家庭认定工作，城市和农村低保标准分别提高到每月610元、395元，累计发放各类救助资金6.51亿元，救助人次达31.4万。学前教育、义务教育、普通高中教育、职业教育全面发展，全市普惠性幼儿园覆盖率提升至66.17%，教育集团发展带动战略深入推进，优质教育资源不断扩充。进贤县、新建区顺利通过义务教育发展基本均衡国家督导评估认定，实现全市全域达标。100%的三级医院开展了医联体建设，组建市级医疗集团7个、县级医共体9个、远程医疗协助网6个、省级专科联盟8个。连续四届获评全国无偿献血先进市。西湖区成功创建国家流动人口基本公共卫生计生服务均等化示范县区。青云谱区通过国家基层中医药工作先进单位评审。全省慢性病示范区创建工作推进现场会在我市召开。就业创业工作扎实推进，创新举办“2018洪城创业故事汇”活动，全市城镇就业率95.1%，零就业家庭就业安置率100%。全民参保工作扎实推进，在全国率先实施社保“统受分办”改革，跨省异地就医直接结算机制进一步完善。文化消费试点好戏连台，文艺创作更加多彩，文化交流更加广泛，在全省率先推出24小时无人值守城市智慧书屋。在全省首创“全民健身一卡通”。南昌代表团在第十五届省运会蝉联金牌总数、奖牌总数、团体总分“三个第一”。

社会治理再上新台阶。“七五”普法宣传教育全面开展，“五区”平安创建活动持续深化，“兴家风、淳民风、正社风”活动成效明显。大力开展扫黑除恶专项斗争，加大治安防控力度，依法严厉打击传销、网络电信诈骗、非法吸收公众存款等涉众型经济犯罪；全力推进信访矛盾化解攻坚战，妥善处置了一批信访维稳突出问题；安全生产工作力度加大，交通运输、建筑工程施工、消防、食品药品等领域安全生产形势稳中向好；统筹做好了村（居）委会换届选举、河道采砂管理、农民工工资“治欠保支”等工作，社会大局保持和谐稳定。我市荣获全国地市级防震减灾工作先进市。南昌县、青山湖区荣获全国法治创建活动先进单位。全国基层社会治理创新的优秀民警代表邱娥国被党中央、国务院授予“改革先锋”称号。

此外，国防动员、人民防空、统计审计、民族宗教、史志档案、气象水文、机关事务、机要保密、智库建设、应急管理、老龄慈善、残疾人、青少年、妇女儿童等各项事业也取得新成绩。

过去一年，我们深入贯彻全面从严治党要求，推动“两学一做”学习教育常态化、制度化，全面开展“五型”政府建设，不断提升政府自身建设水平。依法接受人大及其常委会的监督，自觉接受人民政协的民主监督，主动接受社会和舆论监督，市人大建议、市政协提案办理满意率和基本满意率达到100%。率先在全省开展电视问政和电视报纸媒体聚焦“怕慢假庸散”监督工作，曝光问题159个，处理463人，问责212人及19个单位。着力畅通政府与群众互动渠道，12345政府服务热线反映问题办结率为94.74%，群众满意率为97.41%。严格落实党风廉政建设责任制，自觉接受纪检监察监督，强化审计监督，进一步营造风清气正的政务环境。

各位代表！这些成绩的取得，是党中央、国务院，省委、省政府和市委坚强领导的结果，是市人大、市政协和社会各界监督支持的结果，是全市广大干部群众团结奋斗的结果。在此，我谨代表南昌市人民政府，向全市人民，向所有关心支持南昌发展的同志们、朋友们，表示衷心的感谢，并致以崇高的敬意！

在总结成绩的同时，我们也要清醒地认识到，我市经济社会发展中还存在不少困难和问题：创新型龙头企业不够强，新项目不够大，新旧动能转换不够快；城市功能和品质提升、城乡融合发展还有不少欠账；安全生产存有隐患，污染治理压力较大；基本公共服务均等化水平仍需提高，就业、教育、医疗、住房、养老等方面与群众期待还有差距；一些领域的改革尚未到位，政务服务便民化程度不够；少数干部忧患意识、责任意识、发展意识不强。对此，我们一定高度重视，采取有力措施，切实有效加以解决。

二、2019年主要工作

2019年是中华人民共和国成立70周年，是全面建成小康社会、实现第一个百年奋斗目标的关键之年。面对稳中有变、变中有忧的经济运行形势，今年我市发展面临的不确定性更大、风险挑战更多。我们必须深刻领会我国发展仍处于并将长期处于重要战略机遇期的基本判断及其新的内涵，牢牢把握、全面贯彻“巩固、增强、提升、畅通”八字方针，牢牢把握、全面贯彻新时代江西高质量跨越式发展首要战略的关键环节和战略重点，立足南昌实际、坚持问题导向，着眼于“释放每一份力量、花好每一分钱、做好每一件事”，全面推动经济增长由主要依靠“投资、消费、出口”拉动向同时依靠“改革、创新、开放”提升并重转变，全面推动城市发展由外延式扩张向内涵式提升转变，全面推动城市管理从“大而化之”的粗放型管理向“科学精细”的现代化治理转变，努力在挖掘潜力中做好资源整合、功能融合、力量协同三篇文章，全力推进全市高质量跨越式发展迈出更加坚实的步伐。

今年政府工作的总体要求是：以习近平新时代中国特色社会主义思想为指导，深入贯彻中央经济工作会议、第84次省委常委会、省委十四届七次全会和市委十一届六次全会精神，按照稳中求进工作总基调，全面落实新发展理念，加快推进高质量发展，坚定不移强产业、兴城市、促改革、扩开放、谋创新、优生态、惠民生、抓党建，保持定力，精准发力，持续加力，在大南昌都市圈建设征程中续写发展新篇章，以优异成绩庆祝中华人民共和国成立70周年。

今年经济社会发展的主要预期目标是：地区生产总值增长8.5—9%；财政总收入（省口径）增长6%左右；地方一般公共预算收入增长5%左右；全社会固定资产投资增长10%左右；规模以上工业增加值增长8.5—9%；社会消费品零售总额增长11%左右；实际利用外资增长10%；实际利用内资增长15%；城镇居民人均可支配收入增长8%左右；农村居民人均可支配收入增长8.5%左右；居民消费价格总水平涨幅控制在3%左右；城镇失业登记率控制在4.5%以内；节能减排完成省政府下达任务。

围绕上述目标，我们将重点抓好以下工作：

（一）更加注重产业培育和体系建设，着力增强大南昌都市圈核心实力。大力实施“强攻产业、决战工业”战略，坚持增量、存量并举，以新动能培育为重点，着力构建实体经济、金融、人才、科技“四位一体”发展的现代化产业体系。

加快现代制造业发展。实施总投资1137.6亿元的76个市级重大重点工业项目，年度计划投资277.4亿元。大力开展传统产业优化升级行动。聚焦绿色食品、现代轻纺、新型材料、机电装备制造四大传统优势产业，着力实施技术创新、技术改造、数字化、绿色制造、产能治理、质量品牌、优质企业培育、产业集群优化等八大提升行动。大力推进南昌县食品产业、青山湖区纺织产业两个传统产业优化升级省级分产业试点，统筹推进进贤县医疗器械产业、安义县铝型材产业、南昌县装配式建筑产业等特色优势产业优化升级。深入实施新兴产业倍增工程。重点打造四大战略性新兴支柱产业。汽车和新能源汽车产业：加快推进小蓝江铃股份富山新能源汽车基地和经开区江铃新能源汽车基地等项目，充分运用江铃牌照资源，引进战略合作者，迅速提升产品档次和产业规模，加大新能源汽车应用推广力度。电子信息产业：结合开展全国5G规模组网建设及应用示范和国家“03专项”试点示范，瞄准四个领先，加强产品和服务的研发、示范、应用、推广，加快推进VR产业创新中心建设，着力打造全球VR应用先行示范区；持续发力LED和移动智能终端产业，继续实施硅衬底LED全球合伙人计划，加快竣工兆驰LED外延片和芯片、兆驰1000条LED封装线、乾照光电生产基地、欧菲曲面盖板、欧菲晶润高像素微型摄像头、联创电子高清广角镜头和摄像模组、诺思微系统6吋滤波芯片等项目，继续推进中微半导体MOCVD设备、华勤通讯制造基地、欧菲华光CCM二期、同兴达精密光电等项目，开工建设科莱电子触控显示、维科电池南昌基地、华视光电液晶模块、联决电子FPC产业园等新项目。生物医药产业：以推进中国（南昌）中医药科创城建设为契机，加大中医药产业的扶持力度，着力推进华润与江中合作发展，促进汇仁集团、济民可信等医药产业龙头企业兼并重组、裂变扩张。加快经典名方、配方颗粒、中药二次开发。航空装备产业：抢抓国家强力推进大飞机研发制造的机遇，充分发挥我市机场资源优势，大力发展民用航空制造、通航产业和航空配套服务；加快航空城建设，积极搭建低空空域管理平台、应急救援平台；聚焦军民融合发展，积极承接洪都公司、昌飞公司、航天科技、航天科工等军民融合项目。

加快发展现代服务业。实施总投资2244.6亿元的95个市级重大重点服务业项目，年度计划投资390亿元。生产性服务业方面。推动现代金融、现代物流、工业设计、软件服务外包等生产性服务业向专业化和价值链高端延伸。鼓励设立工业设计企业和服务中心，培育研发设计、科技成果交易市场，推广施工设备、生产线等融资租赁业态。以降低城市物流综合成本为目标，加快现代物流业发展。依托昌北国际机场智慧空港物流中心，推进航空货运发展。依托向塘物流港、昌北省域综合交通枢纽、南昌综保区、铁路物流基地等平台，打造国际陆港。大力发展城市高效配送体系建设，积极开展全省城市共同配送试点。加快江西尧泰风快平台和马力冷运物流信息平台发展，推进全国智慧物流配送示范城市建设。生活性服务业方面。聚焦打造红谷滩中央商务区、八一广场中山路市级商业中心，加快推进现代出版传媒产业基地、苏宁广场、华侨城文化旅游综合体、华润万象城、绳金塔民俗风情街等重大项目建设；推进王府井城市综合体、新洪城大市场、绿地井象风情商业街、万寿宫历史文化街区、江西国际影视文化城等项目建成开业。大力引进盒马鲜生、网易考拉等引领性消费品牌，推动消费升级。以南昌综保区为核心，加快建设跨境电商线上综合服务平台、国际邮快件监管中心，力争三年内率先在跨境电商进口供应链、跨境电商B2B和B2C出口、跨境电商服务贸易等三类业务模式取得创新突破。力争2019年全市电子商务相关交易额突破4000亿元，增长33%以上。启动国家全域旅游示范区创建工作，完善全域旅游发展规划，全面梳理南昌文化旅游资源，深入挖掘、提升八一文化、豫章文化、街巷文化、赣商文化以及八大山人、王阳明等历史文化名人文化内涵，建立全域旅游产品谱系和业态体系。整合各方资源，构建全要素区域集散中心，强化区域性旅游目的地辐射集聚力。加快汉代海昏侯国遗址公园博物馆、海昏大道、展示服务中心三大项目建设，确保年内高标准开园。积极推动万达文化旅游城创建5A景区，推动湾里区创建国家全域旅游示范区和国家级旅游度假区。

加快培育新产业、新业态、新模式。大力发展物联网、大数据、云计算、人工智能等前沿科技产业，加大前沿科技向实体经济渗透力度，推动产业发展向智能化、数字化转型。树立跨界融合、三产联动思维，培育“工业+”“服务业+”“农业+”等新业态、新模式，力促传统行业与网络化、智能化、便利化、个性化消费趋势深度融合，加速一二三产在更细领域更深层次融合发展。

加大园区承载产业力度。优化园区布局。坚持以全市产业布局规划为引领，立足资源禀赋差异化，明确首位产业和主攻产业，引导各园区科学有序、错位互补、协调发展。按照“资源整合、优势互补、发展互动”的思路，大力推行飞地经济发展模式，着力推进高新区与进贤县、青山湖区与进贤县、经开区（临空区）和新建区、经开区（临空区）和综保区合作建设特色产业基地。完善园区配套。将园区基础设施建设纳入城市统一规划、统筹建设。加快推进电力、燃气、供热、供水、通信、道路、消防、防汛、人防、治污等基础设施建设。支持园区打造产业邻里中心，加快园区“三房”建设。提升园区服务。以“新九通一平”建设为抓手，加强园区政务服务、公共信息、研发设计、检验检测、技术转移、融资服务、物流配送等服务功能，打造一体化综合型服务平台，探索智慧园区建设，努力让企业不出园区就能享受到各项服务。推动体制机制创新。以“权力下放、资金下沉、重心下移、监管跟上、考核精细”为主线，推动体制机制创新，全面激发园区发展活力。推动园区落实国家级开发区全链审批赋权清单，制定并公布省级开发区全链审批赋权清单，推动国家级、省级开发区享受相应经济管理权限。依法简化审批事项申报环节，简化审批管理层级，提升审批效率。落实深化开发区干部人事和薪酬制度改革暂行办法，形成岗位能上能下、人员能进能出和以岗定薪、按绩计酬的人事薪酬制度。树立“亩产论英雄”导向，对工业园区实施差异化的激励和淘汰考核机制，提高土地产出率，加快处置“僵尸企业”“僵尸项目”，为新产业、新动能腾出空间。

加大金融支撑产业力度。全力激发金融发展活力，支持赣江新区开展绿色金融改革试点，加快红谷滩全省金融商务区发展，大力引进全国性、区域性金融机构和外资金融机构来昌设立区域性总部，积极引进和发展各类金融服务机构。着力扩大信贷规模，努力克服资金面偏紧和政策面趋严的困难，强化与开发性、政策性、商业性金融机构战略合作，全力保障全市重点基础设施、“一核两重”产业和乡村振兴项目融资需求，引导更多“金融活水”流向实体经济，助力企业特别是民营企业跨越市场的“冰山”、融资的“高山”、转型的“火山”。奋力推进企业上市“映山红行动”，进一步提高直接融资比重，壮大资本市场“南昌板块”。大力实施科技金融推进工程，坚持围绕产业链布局科技链，聚焦科技链完善资金链，推动科技与金融相互促进、良性循环。

加大人才服务产业力度。营造氛围聚人才。加大“人才新政”宣传力度，让人才从政策“新意”中看到南昌“诚意”。持之以恒抓好人才新政的实施，确保100亿人才资金每一分都真正用在引进人才、培育人才上。搭建平台引人才。统筹推进“洪”字号系列人才工程，重点围绕汽车和新能源汽车、电子信息、生物医药、航空、现代金融等产业精准引育。吸引国家大院大所、知名高校、上市公司及大型企业在昌设立研发机构。力争5年内全市建成100家院士工作站、博士科研工作站、国家工程技术中心、省级以上重点实验室等科研创新平台。配套举措为人才。全面落实“天下英雄城•聚天下英才”行动计划，加快形成重点突出、层次分明、覆盖广泛、精准有效的“1+N”政策体系，着力解决事业和待遇两大人才最关心的问题。

加大科技推动产业力度。做优创新布局。着力推进赣江两岸科创大走廊建设以及江西鄱阳湖国家自主创新示范区创建工作，加快建设南昌航空科创城、中国（南昌）中医药科创城、南昌国家大学科技城以及红谷滩科创小镇，积极推进创新型县区、开发区建设。做强创新主体。实施企业研发机构建设攻坚行动计划和高新技术企业量质“双提升”工程，力争2020年全市规模以上工业企业和高新技术企业建立研发机构的比例分别达到30%、50%，到2023年均达到80%。做实科研平台。支持企业与省内外高校院所共建产学研合作平台，重点推进中科院苏州纳米所南昌研究院、哈工大机器人南昌智能制造研究院、中山大学南昌产业研究院建设，加快筹建宁波智能制造产业研究院南昌分院、南昌同济大学江铃汽车创新研究院。做好成果转化。成立科技成果转化中心，搭建南昌科技广场线上线下科技成果交易平台，设立科技成果数据库，鼓励企业和高校院所共享科研成果和知识产权信息。提高中国（南昌）知识产权保护中心运营效益，着力打造全国一流的知识产权快速审查、快速确权、快速保护功能性平台。

（二）更加注重城市更新和区域协同，着力提升大南昌都市圈整体功能品质。

按照统筹融合的思维和城市更新的理念，以全省城市功能与品质提升三年行动计划为统领，以大南昌都市圈区域协同发展为重点，以“美丽南昌•幸福家园”环境综合整治行动为抓手，树立富规划、精建设、细管理的理念，全力做好“规、建、管、养、监”五字文章，推动城市品质和功能的全面提升。

突出规划引领。强化编制力量。结合机构改革，组建专门的规划编制单位，整合专业各具专长、经验丰富的规划编制力量，统筹推进各类规划的编制工作。提升规划水平。吸引国际国内高水平的规划团队参与我市规划编制工作，不断加快国土空间规划和各类专项规划以及重点区域、重要节点的规划编制和城市设计工作。重点抓好大南昌都市圈规划一体化、高铁东站周边地区控制性详规和扬子洲地区空间规划的编制工作。

坚持高水平建设。实施总投资1547.7亿元的67个城市建设重大重点项目，年度计划投资268亿元。大力完善基础设施。加快推进昌吉赣客专、昌景黄客专建设，积极做好昌九客专、昌北国际机场三期扩建工程和西二环高速公路前期工作，并力争开工，加快构建大南昌都市圈同城化交通体系。推动地铁2、3、4号线建设“三线并进”，确保实现2号线后通段开通运营；实现3号线“洞通”、4号线7座车站主体封顶，积极争取国家支持，着力推动1号线北延至机场段、2号线东延至高铁南昌东站工程规划申报工作。继续推进城市“三环”和“十横十纵”干线路网建设，启动二七路过江隧道、九龙湖过江通道、九洲高架快速路东延、城市二环北段等工程前期工作；争取长江委、省水利厅支持，力争沿江中北快速路年底前动工；新开工建设丹霞路、桃新大道、赣新大道等工程；继续推进艾溪湖隧道、昌东大道改造、英雄大道改造等工程建设，确保昌西大道基本建成，洪都大道、昌南大道、昌九大道等快速路竣工通车，实现城市“一环”和“三环”闭环。结合城市综合管廊建设，启动青山湖西岸片区基础设施建设，加快推进洛阳东路综合管廊、九龙湖新城综合管廊一期工程。继续推进旧改棚改。按照“拆、改、提、留、建”的思路，继续推进旧城区改造，稳步推进27278套棚户区改造；加快推进中心城区集中安置小区建设；制定出台“家改居”实施办法，加快推进老旧社区环境整治工作。着力丰富文化内涵。深入挖掘西湖区进贤仓、青云谱区洪都老工业区、东湖区八一起义系列等历史文化资源，推动旧城改造和历史文化相互融合，使城市的“一路、一街、一景、一亭、一园”都彰显历史记忆、延续历史文脉。

加强精细化管养。实施总投资265亿元56个市级重大重点城市管理项目，年度计划投资114亿元。聚焦全国文明城市复查迎检的重点工作和薄弱环节，全面推进城市功能与品质提升八大行动，全力实现“美丽南昌•幸福家园”环境综合整治“三年上台阶”目标。大力开展“治脏”行动，重点推进“以克论净”的“马路本色”行动、城乡统筹的垃圾分类行动，加大建筑工地、住宅小区环境卫生治理力度。大力开展“治乱”行动，重点推进“五车六乱”专项整治行动，加大建筑立面和户外广告、老旧农贸市场改造力度，加大占道摊点、违搭乱建规范力度。大力开展“治堵”行动，综合运用好“加、减、乘、除”综合法则，破解城市交通拥堵难题，进一步加大交通基础设施投入，优化完善城市功能布局，大力发展公共交通，推进精细城市交通治理，重点完成69个新老交通堵点的治理，稳步推进“互联网+交通信号控制”和立体监控体系建设，全力打造“智慧南昌交通”。大力开展“功能修补”行动，重点推进污水处理、市政设施维护、海绵城市等工作。大力开展“生态修复”行动，重点推进黑臭水体长治久清、“300米见绿，500米见园”系统建设。大力开展“特色彰显”行动，重点推进水景观建设、历史文化街区建设；大力开展“亮化美化”行动，重点推进“一江、三河、九湖”景观亮化打造。大力开展“治理创新”行动，结合机构改革，厘清各方责任，全面理顺城市管养“条条、条块、块块”职责权限，落实属地责任；加快实施管养分离，推动城市管理由“以费养人”向“以费养事”转变，加大环卫保洁、市政设施维护、绿化养护、城市照明等领域的市场化改革力度。

强化运行监测。以智慧城市建设为抓手，结合大数据管理局组建、“03专项”和“5G”试点，采用“互联网+”“物联网+”等手段，推动城市管理、城市交通、治安消防、防震减灾、应急管理、国防动员等功能融合、数据共享、分口应用，加快打造“城市大脑”，逐步实现对整座城市全方位、全过程动态监测。探索完善城市管养监管新模式，引进第三方专业机构，强化监测体系建设，通过大数据分析和专业机构评估，对各级各部门各单位履行城市管养职责进行科学评价，严格做到按责考核、按质取费、奖优罚劣，实现城市管养的常态化、长效化、精细化。

（三）更加注重改革攻坚和开放提升，着力激发大南昌都市圈发展活力。

强化重点改革攻坚，提升政府运行效率和经济社会发展活力。深化市县政府机构改革，加快落实调整组建、转隶安置、集中办公、新机构挂牌、资产档案划转交接等工作。倡导“不为说不找理由，只为说行想办法”的工作理念，以“一网通办、一次不跑、最多跑一次”为重点，深化审批服务便民化改革；以创新“六多合一”模式为龙头，深化建筑工程领域“四个联合”、商事登记领域“证照分离、证照联办”改革。编制南昌政务服务“百事通”，推广政务服务“自助办”，提升南昌政务服务温度，打造南昌政务服务品牌。持续推进“互联网+不动产登记”建设，深入推进“不见面”审批，压缩办结时限。深化国资国企改革，加快市属经营性国有资产集中统一监管，推动市属国有企业进一步优化重组。深化投融资体制改革，拓宽融资渠道，科学谋划、分类梳理一批公益性、准公益性、市场化的项目，破解产业发展、城市建设、乡村振兴、生态治理四大方面投融资难题。坚决打好防范化解重大风险攻坚战，积极稳妥处理地方政府债务风险。继续深化智慧市场监管、税务便利化、医药卫生体制等各项改革。

提升双向开放层次，拓展经济腹地和发展空间。扩大政策优势。积极借鉴上海自贸区先行先试经验，完善开放型经济体制机制。进一步完善外贸支持政策，缩短贸易类外贸企业出口退税周期，深入推进国际贸易“单一窗口”国家标准版推广应用。强化平台功能。围绕“进境与沿海同价到港，出境与沿海同价起运，通关与沿海同等效率”的目标，强化南昌综合保税区与机场、码头、铁路等口岸平台的协调互动，打造全国首个“内陆服务平台型”综保区；推动口岸功能提升，持续推进空港、无水港建设和进出口通关能力建设，加快推进南昌铁路口岸二期工程建设，促进南昌海铁联运和中欧班列健康快速发展，加快推进指定口岸和邮件快件监管中心申报建设，持续提升跨境贸易便利化水平。加强区域合作。争取省里出台大南昌都市圈“1+5”规划及相关支持政策，充分发挥省会集聚辐射作用，携手九江、抚州、丰樟高、鄱余万等地区做大做强“引领圈”，增强对全省发展的支撑力和带动力。进一步深化与长江中游城市群、闽浙赣皖福州协作区、泛珠三角城市群城市交流合作，主动对接粤港澳大湾区、海南自贸区（自贸港）、海西经济区和长三角一体化发展，着力承接先进制造业梯度转移。积极参与“一带一路”建设，加快我市国际经济技术合作的发展升级，带动技术、产品、设备和服务“走出去”，促进外经外贸融合发展。实施招大引强工程。用好进口博览会、中博会、赣商大会、赣港（深）会、赣台会、世界VR产业大会、国际中医药博览会等会展优质平台，以“三请三回”“三企入赣”活动为抓手，积极开展主题、专题招商活动和走访对接，依托重点产业全景图，围绕上下游产业链拓展相关配套产业，加强对重点区域、重点行业、龙头企业、重点客商的精准招商。

（四）更加注重农业农村优先和城乡融合发展，着力推动大南昌都市圈乡村振兴。

坚决打赢精准脱贫攻坚战。紧盯年度8200多名贫困人口脱贫任务，按照高质量脱贫的目标要求，继续巩固提升已退出的80个贫困村和已脱贫人口的脱贫成效和质量，在全面落实“两不愁、三保障”政策的基础上，精准落实产业扶贫、教育扶贫、健康扶贫、保障扶贫等措施。采取相应方式帮助收入水平略高于建档立卡贫困户的群体增收致富，防止出现脱贫攻坚的“夹心层”。大力开展脱贫攻坚“感恩行动”，教育引导贫困群众感恩奋进、勤劳致富。

推动农村产业兴旺。实施总投资205亿元的13个市级重大重点农业及城乡统筹项目，年度计划投资53.7亿元。全面落实“藏粮于地、藏粮于技”战略，坚决守住耕地红线，加快推进高标准农田建设，大力实施粮安工程，确保农产品有效供给。坚持质量兴农、绿色兴农，加快构建现代化农业产业体系，确保中国云厨南昌全域产业园、铭宸农业科技园竣工建成；以南昌绿谷为核心，全力打造一大批农文旅深度融合的特色小镇，促进民俗民宿产业、美丽经济、生态景区、康养产业等观光休闲农业快速发展；加快推进25个市级田园综合体、50个农业产业化联合体、100个现代农业园区建设，形成示范带动效应，加速南昌现代农业一二三产融合发展。重点培养扶持本地龙头企业，推进“一村一品”基地建设，接续扶持100个左右新型农业经营主体。切实抓好春秋防疫、春夏防汛、农产品流通防残毒等基础工作。

加快美丽宜居乡村建设。大力实施农村人居环境整治三年行动，深入推进农村“五拆五清”工作，完成年度2000个左右村点的建设任务，实现“三年扫一遍”的目标。持续改善贫困村村庄环境和贫困户家居环境。大力开展“四精”美丽乡村试点创建，加快推进安义县、湾里区省级全域美丽乡村示范及各县区秀美乡村示范线路建设。按照“全域规划、多规合一，因地制宜、富有特色，留住乡愁、留下记忆”的思路，支持建设一批特色示范小镇、“五位一体”新农村和农村新户型建设示范点。稳步推进绿色殡葬改革，破除陈规陋习，培育文明乡风。

稳妥深化农村改革。巩固和完善农村基本经营制度，深化农村承包地“三权”分置、“两权”抵押贷款试点、农村宅基地、农村集体产权制度改革。继续抓好“财政惠农信贷通”，积极破解农业经营主体融资难题。挖掘盘活后备耕地开垦资源，抓好“旱改水”项目实施，着力解决耕地占补平衡问题。因地制宜发展土地整合型、股份合作型、资源开发型、物业经营型、乡村服务型等多种集体经济形式，力争2019年全部消灭村集体经济空壳村。

（五）更加注重生态优先和绿色发展，着力提升大南昌都市圈区域特色优势。

持续加大污染防治力度。坚决抓好中央和省环境保护督察“回头看”反馈问题整改落实。严格执行生态保护红线，扎实开展生态示范创建和自然保护区监管。打好蓝天保卫战，持续抓好“四尘三烟三气两禁”专项整治，让蓝天永驻。打好碧水保卫战，加强饮用水水源地环境监管、工业企业水污染防治、污水处理厂建设和管理、农业农村水污染治理、“河湖长制”水质考核，全力打造“东方水城•中国水都•鄱湖明珠”品牌，让碧水长流。打好净土保卫战，扎实推进土壤环境质量监测、污染详查新增土壤污染防控，让净土芬芳。打好青山保卫战，全面推行“林长制”，加强梅岭等重点区域松材线虫病预防和除治，持续开展造林“绿化、美化、彩化、珍贵化”建设，强化森林防火工作，让青山常绿。打好环保执法持久战，加大定期执法、联动执法和信访投诉执法力度，健全行政执法与刑事司法衔接配合机制，打击环境违法行为。力争全市空气质量优良率超过85%，PM2.5年均浓度保持空气质量二级标准，城市集中式饮用水源地水质达标率达100%，赣江、抚河断面水质达标率大于92%。

持续加大生态建设力度。深入推进国家生态文明试验区建设，健全自然资源确权登记、价值实现、离任审计、责任追究等制度，用最严格制度最严密法治为生态环境建设保驾护航。大力发展绿色产业，推动生态要素向生产要素、生态财富向物质财富转变，打通绿水青山到金山银山的转化通道。严格执行生态保护红线，扎实开展生态示范创建和自然保护区监管，高品质建设南昌山水田林湖草生命共同体示范工程。督促企业落实环保主体责任，严格遵守环保法律法规，履行环保资金投入、物资保障、措施强化和应急处置等方面的义务。鼓励支持群众自治组织、社会组织和环保志愿者开展环保工作，倡导简约适度、绿色低碳的生活方式，让“保护环境从我做起”成为全社会的共识共为。

（六）更加注重民生兜底和优化供给，着力夯实大南昌都市圈全面小康基础。

健全完善社会保障体系。积极开展援企稳岗“护航行动”，建立困难人群的就业帮扶机制，确保“零就业家庭动态清零”。重点解决好高校毕业生、农民工、退役军人等群体就业问题，进一步完善青年创业服务体系，举办第二届“洪城工匠”培养选树活动，持续激发“大众创业、万众创新”活力。坚持“房住不炒”原则，强化市场监测和调控，促进房地产市场平稳健康运行。加强住房保障，优先保障困难职工住房需求，对低保、低收入住房困难家庭实现应保尽保。进一步完善社保业务标准化信息化建设，全面深入实施全民参保计划，稳步提高城乡基本养老、基本医保、城乡低保等补助标准，继续完善基本医保跨省异地就医住院费用直接结算机制。深入抓好城镇贫困群众脱贫解困工作。稳步提升特困人员供养水平，建立健全“老弱病残幼”特殊群众关爱服务体系，加快市老年养护院、市老年活动中心、市儿童福利新院、市妇女儿童社会服务中心建设。

扩大公共服务优质供给。实施总投资203亿元的45个市级重大重点社会事业项目，年度计划投资68.7亿元。坚持教育优先发展战略，继续推进第三期学前教育行动计划，推进义务教育由基本均衡向优质均衡发展，稳妥实施好教师编外聘用和“县管校用”机制，深入实施高中阶段教育普及攻坚计划和第二期特殊教育提升计划，加快推进朝阳中学、十九中初中部红角洲校区等城乡中小学校园工程建设。扎实推进县（区）中职达标校建设。支持豫章师范学院做大做强。推进健康南昌建设，加快推进医联体建设，建立健全现代医院管理制度，深化“互联网+医疗健康”发展，构建优质高效的医疗服务体系。科学处置各类传染病疫情。持续推进社会主义核心价值观体系建设，大力实施南昌文化云、城市智慧书屋、基层综合文化服务中心等文化工程，鼓励发展私人博物馆、美术馆、展示馆；支持创作文艺精品作品，开展送书、送电影、送戏等文化下乡进社区活动，办好庆祝新中国成立70周年系列展览展演。办好“天下英雄城，激情马拉松”等全民健身活动，做好“八一南昌”职业赛事服务保障工作，把南昌打造成为全国具有较大影响力的体育名城。

探索社区发展新模式。按照“党建引领、社区主体、属地负责、部门协同、因地制宜、分类实施、示范先行、统筹推进”的原则，探索推进“1+5+X”社区邻里中心建设。就是每个社区规划配置幼儿园（幼教点）、居家（社区）养老服务中心、基层医疗卫生服务点、邻里生鲜便利店、综合性文化（体育）中心5类服务功能，另外，因地制宜每个社区拓展若干个其他业态。形成“1+5+X”社区邻里中心建设标准、管理规范及运营机制等一套规范体系，绘制一张全市社区邻里中心分布蓝图，建成一批布局合理、业态齐备、功能完善、服务有序的普惠型社区邻里中心。力争到2019年底试点建设42个，到2020年底推广建设150个，到2021年底建成300个左右，实现中心城区全覆盖。同时，因地制宜制定农村版标准规范体系，完善一批中心村公共服务设施。

加强和创新社会治理。深化法治南昌建设，完善行政执法和监督体系，进一步健全和完善公共法律服务体系。抓好普法宣传教育，深入实施“法律明白人”工程，充分发挥市民公约、乡规民约等社会规范作用，抓好宗教领域突出问题整改工作，依法规范宗教事务，推进移风易俗，提高社会文明程度。加快“雪亮工程”建设。探索城市管理和社会综合治理“两网合一”融合治理模式。进一步加强信访法治化建设，积极探索新时代“枫桥经验”南昌信访实践，妥善化解重点领域信访问题。深入推进扫黑除恶专项斗争，着力铲除黑恶势力滋生土壤。持续开展打击传销、电信网络诈骗、非法集资等涉众型经济犯罪，“食药环”类犯罪和偷盗抢、黄赌毒等违法犯罪活动。推进安全生产社会化、法治化、智能化、专业化，坚决遏制重特大安全事故。深入推进国家食品安全示范城市创建。完善应急管理和防灾减灾救灾体制机制，为人民群众提供更加可靠的安全保障。做好全国第四次经济普查工作。扎实做好国防动员、国防教育、国家安全、人民防空和退役军人事务工作，争创全国双拥模范城“九连冠”。

全力以赴办好民生实事。坚持“保基本、守底线、抓重点”的原则，积极筹集各类资金500亿元以上，实施100件左右的民生实事，不断在幼有所育、学有所教、劳有所得、病有所医、老有所养、住有所居、弱有所扶上取得新进展。重点实施总投资237亿元的44个市级重大重点民生实事项目，年度计划投资62亿元。其中，根据市委关于政府民生实事项目人大代表票决制决定，按照“充分体现民意、注重全面统筹、力求务实高效”的要求，通过广泛征集、层层筛选，遴选了12件候选项目。（1）教育惠民便民工程。①全市义务教育阶段学校实施校内课后服务；②市属中小学运动场地免费向社会开放。（2）农村饮水安全巩固提升工程。2019年在南昌县、进贤县、安义县、新建区范围实施农村饮水安全提升工程，解决20万人农村居民饮水安全问题。（3）水系综合治理工程。2019年完成幸福中河、义坊河水系整治项目。（4）食用农产品安全保障水平提升工程。2019年试点建立全市食用农产品电子化追溯体系；提升全市食用农产品检验检测能力。（5）中心城区既有住宅电梯加装工程。对符合条件且业主意愿一致的住宅开展加装电梯试点工作。（6）农贸市场建设改造工程。2019年全市开工新建改造8个城乡农贸市场。（7）老旧燃气管网改造工程。2019年改造市区及市郊范围老旧管网铸铁管103千米，覆盖12000户，杜绝燃气泄漏隐患，提升稳定供气能力。（8）老旧社区环境整治工程。2019年完成21个老旧社区环境整治。（9）推动“厕所革命”。2019年新建装配式移动公厕118座，改造公厕67座。（10）加强消防基础设施建设。2019年增补既有道路1094个市政消火栓建设，新建道路同步建设市政消火栓。（11）农村公路建设改造工程。2019年完成80公里农村公路通自然村、50公里农村公路改造、5座农村公路危桥改造。（12）方便市民出行，实施公交优先战略。①购置车辆120台，新增公交线路4条以上；②全市持证残疾人享受免费乘坐市内公共交通。上述12件民生实事项目，现提请各位代表审议。对票选的民生实事项目，市政府将加强组织领导、严格责任考核、强化统筹协同、狠抓工作落实，并接受市人大代表和社会各界监督检查，确保实事办好、好事办实。

与此同时，大力支持工、青、妇、残联、红十字会等群团组织建设，发挥社会组织的积极作用；做好民族、外事、侨务、税务、地震、气象、地质、供销、档案、史志、老龄、慈善、援疆、机关事务、机要保密等工作。

三、加强政府自身建设

各位代表！新时代新使命对政府工作提出了新的更高要求。我们将全力建设忠诚、创新、担当、服务、过硬“五型”政府，着力提升政府治理体系和治理能力现代化水平。

建设忠诚型政府。旗帜鲜明讲政治，对党绝对忠诚，牢固树立“四个意识”、坚定“四个自信”、做到“两个维护”，自觉在思想上政治上行动上同以习近平同志为核心的党中央保持高度一致。强化学习重运用，坚持用习近平新时代中国特色社会主义思想武装头脑、指导实践、推动工作，时时处处事事贯彻新发展理念，立足于从更高层次贯彻习近平总书记对江西工作的重要要求，坚定不移推动党中央、国务院，省委、省政府和市委的决策部署在南昌落地生效。

建设创新型政府。紧紧扭住做大做强做优大南昌都市圈的薄弱环节和重点工作，大兴调研之风，加强新型智库建设，坚持问题导向补齐能力“短板”，提升各项事业改革攻坚、开放提升、创新突破的操作水平；大兴开拓之风，强化创新创业精神，努力在各项工作中创亮点争进位；大兴协同之风，加快全市范围内跨区域、跨部门、跨层级的资源整合、功能融合、力量协同，着力推动全市经济社会高质量跨越式发展。

建设担当型政府。坚决贯彻市委的决策部署，认真执行人大及其常委会的决定，及时办理人大代表建议、议案和政协委员提案、协商建议，自觉主动接受人大及其常委会、政协、监察、司法、审计、社会、舆论等各方面监督。大力倡导“事事马上办、人人钉钉子、个个敢担当”的工作作风，对重点项目、重要工作，做到“责任到人、时限到点、考核到位”。坚持“三个区分开来”，为担当者担当负责，让想为者敢为有为，凝聚英雄城强大正能量。

建设服务型政府。抓紧编制机构改革后新的权责清单，做到“法无授权不可为、法定职责必须为”。着力打造“四最”营商环境，构建亲清新型政商关系，支持民营经济健康发展，为企业提供无需不扰、有求必应的“店小二”服务。持续开展精准扶贫、挂点帮扶、矛盾调处等基层大走访活动，用好12345政府服务热线、南昌问政现场、聚焦“怕慢假庸散”、政务微博、政务微信等平台，做到民有所呼、政有所应。

建设过硬型政府。推动全面从严治党不断向纵深发展，坚决全面彻底肃清苏荣案余毒，着力营造政府系统风清气正的政治生态。持续开展作风建设“八大”举措，坚决克服形式主义、官僚主义。改进政务督查，严控总量和频次，切实减轻基层负担。带头过紧日子，严管公共资金，严管公共资源交易，严管国有资产资本，严控“三公”经费和一般性支出，把有限的资源和财力用在推动发展、改善民生上。

各位代表！新时代是奋斗者的时代。让我们紧密团结在以习近平同志为核心的党中央周围，在省委、省政府和市委的坚强领导下，不忘初心、牢记使命，脚踏实地、埋头苦干，全力以赴做大做强做优大南昌都市圈，加快打造富裕美丽幸福现代化江西“南昌样板”，为共绘新时代江西物华天宝人杰地灵新画卷作出新的更大贡献，以优异成绩庆祝中华人民共和国成立70周年！
